# Hallazgos similares al COVID-19 en un caso fatal de neumonía intersticial descamativa asociada con glomerulonefritis por IgA en una niña de 13 meses de edad

**DOI:** 10.1159/000516149

**Published:** 2021-04-12

**Authors:** Simona Gurzu, Catalin-Bogdan Satala, Lorena Elena Melit, Adrian Streinu-Cercel, Dan Otelea, Brandusa Capalna, Claudiu Ioan Puiac, Janos Szederjesi, Ioan Jung

**Affiliations:** 1 ^a^Departamento de Patología, Universidad George Emil Palade de Medicina, Farmacia, Ciencias y Tecnología, Targu-Mures, Rumania; 2 ^b^Departamento de Patología, Hospital de Emergencias Clínicas del Condado, Targu-Mures, Rumania; 3 ^c^Departmento de Microscopía, Centro de Investigación de la Universidad de Medicina, Farmacia, Ciencias y Tecnología, Targu-Mures, Rumania; 4 ^d^Departamento de Pediatría, Hospital de Emergencias Clínicas del Condado, Targu-Mures, Rumania; 5 ^e^Departamento de Pediatría, Universidad George Emil Palade de Medicina, Farmacia, Ciencias y Tecnología, Targu-Mures, Rumania; 6 ^f^Instituto Nacional para Enfermedades Infecciosas «Matei Bals», Bucarest, Rumania; 7 ^g^Departamento de Cuidados Intensivos, Universidad George Emil Palade de Medicina, Farmacia, Ciencias y Tecnología, Targu-Mures, Rumania

**Keywords:** Infante, Neumonía intersticial, Autopsia, SARS, COVID, Glomerulopatía, IgA, Berger

## Abstract

En la era del COVID-19, es usual sospechar que cualquier paciente con síndrome respiratorio agudo grave (SARS) esté asociado con una infección por SARS-CoV-2. El objetivo de este artículo es presentar un caso de neumonía similar al COVID, con evolución fatal. Los aspectos clínicos se correlacionan con los hallazgos en la autopsia y se discuten en el contexto de los datos más recientes en la literatura médica. Una niña de 13 meses de edad ingresó a la sala de emergencias con dificultad respiratoria aguda y opacidades pulmonares bilaterales con apariencia de vidrio molido, además del pulmón izquierdo casi completamente opacificado. El estado de la paciente se deterioró súbitamente, y se confirmó la muerte 3 h después de la admisión. En la autopsia se diagnosticó neumonía intersticial descamativa grave, y se asoció con glomerulonefritis por IgA, un hallazgo poco usual. No se detectó infección por SARS-CoV-2 en el parénquima pulmonar mediante RT-PCR. Éste es un caso muy inusual de deterioro rápido de un infante con neumonía intersticial descamativa (NID) idiopática y afectación multiorgánica. Con base en tinciones inmunohistoquímicas, proponemos la hipótesis de que, en la NID, las membranas hialinas surgen de neumocitos descamados necrotizantes. En la era de COVID-19, tales casos son extremadamente difíciles de diagnosticar, y pueden semejar las lesiones pulmonares inducidas por el SARS-CoV-2. Esta pauta de formación de membrana hialina podría explicar la falta de respuesta a la terapia con oxígeno. El presente caso resalta la importancia de la autopsia en estos casos complicados.

## Introducción

La neumonía intersticial descamativa (NID) es un padecimiento raro: solamente se han reportado 362 casos en adultos, la mayoría entre fumadores [1]. En niños, no había consenso sobre su etiología y mecanismo patológico en los 41 artículos escritos en inglés hallados en la literatura hasta 2020 [1, 2, 3]. Hasta donde sabemos, solamente tres de los casos de NID reportados en niños presentaron insuficiencia renal asociada [4], y ninguno mostró un deterioro rápido y fatal en pacientes menores de 2 años.

Se cree que la NID en los niños es causada por un defecto congénito en el metabolismo del surfactante, el cual se asocia con síntomas respiratorios que progresan rápidamente y con un impacto negativo en el crecimiento [2, 3]. Se cree que su patogénesis está mediada por la inmunidad [4] y que predispone a la fibrosis pulmonar [3].

En este artículo presentamos un caso inusual de NID diagnosticado en una niña de 13 meses de edad, quien murió algunas horas después de su ingreso al hospital. Varios aspectos hacen de éste un caso único en la literatura. En primer lugar, comenzó en una bebé aparentemente saludable, con un deterioro rápido en el estado de su salud. En segundo lugar, la asociación con glomerulonefritis a tan temprana edad no se ha descrito aún en la literatura. Luego, puesto que el caso se diagnosticó durante la pandemia de COVID (enfermedad por coronavirus 2019), y el cuadro histológico de los signos clínicos y morfológicos se correspondió con un síndrome respiratorio agudo grave (SARS), se sospechó de una infección por SARS-CoV-2; sin embargo, ésta no se confirmó por repetidos ensayos de RT-PCR. El caso resalta algunos de los desafíos que los departamentos de pediatría podrían enfrentar muy pronto, así como la importancia de la autopsia en casos difíciles, que pudieran semejar una infección por SARS-CoV-2. Junto con la presentación del caso, se ofrece una revisión de la literatura reciente con respecto a las posibles características clínicas y morfológicas inducidas por el COVID, junto con un paradigma sobre la formación de membrana hialina en casos de NID. Este último hecho podría explicar la poca respuesta a la terapia con oxígeno en casos inducidos por COVID y casos con signos similares.

Hasta donde sabemos, el presente es el cuarto caso de NID con afectación renal descrito en niños en la literatura en inglés, y el primero con evolución fatal, posiblemente como resultado de una infección asociada, similar a la causada por el SARS-CoV. Se obtuvo el consentimiento informado de la madre para la publicación de este caso.

## Reporte de caso

Durante la guardia nocturna en nuestra sala de emergencias, una niña de 13 meses de edad fue remitida a nuestra institución con tos, escalofríos, dificultad respiratoria grave y fiebre (38 °C). De acuerdo con el historial médico obtenido de la madre, no se reportaron anomalías perinatales o posnatales del crecimiento o el desarrollo. El deterioro comenzó algunas horas antes del presente ingreso, y se negó un posible contacto con personas infectadas con SARS-CoV-2.

El examen físico reveló una piel y mucosas pálidas, y una bebé hipotónica con hepatoesplenomegalia, taquipnea, taquicardia y una saturación de oxígeno de 80%. La disnea grave requirió el uso de emergencia de una máscara facial de oxígeno. Se practicó la intubación unos minutos después. No se identificaron anomalías en el ECG ni en el ultrasonido abdominal.

El análisis sanguíneo indicó deshidratación y anemia grave − con un nivel muy bajo de hemoglobina sérica (1.5 g/dL) y un hematocrito bajo (6.7%). La uremia leve y los niveles ligeramente disminuidos de creatinina sérica (0.36 mg/dL) se consideraron indicadores de deshidratación. También se observó un nivel ligeramente aumentado de proteína C-reactiva sérica (14.6 mg/L) (Tabla 1). El análisis de orina mostró leucocituria, pero no bacteriuria.

**Fig. 1:**
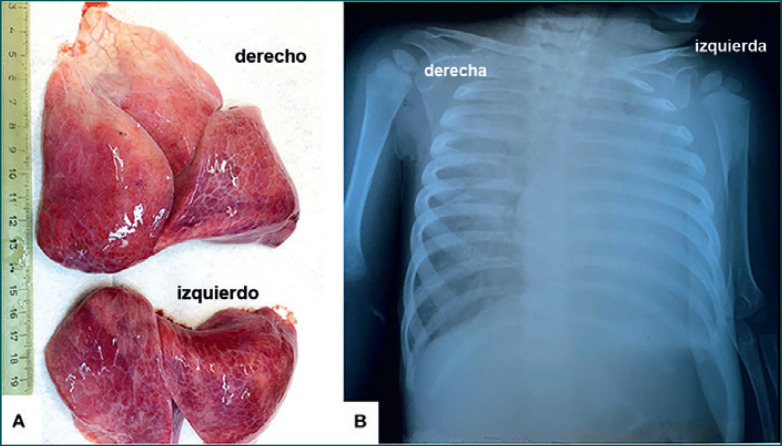
En una niña de 13 meses de edad, la distelectasia pulmonar bilateral grave, revelada por examen post mortem (**A**), corresponde a la imagen por Rx, que muestra opacidades con aspecto de vidrio molido en el pulmón derecho, y opacidad difusa en el pulmón izquierdo (**B**).

Una radiografía torácica mostró la opacidad casi completa del pulmón izquierdo e infiltrados espaciados en el pulmón derecho (Figura 1). A pesar de la administración de bolos intravenosos de fluidos y del cuidado de soporte − incluyendo terapia con oxígeno −, se confirmó la muerte 3 h después del ingreso al hospital.

Debido al súbito compromiso respiratorio asociado con los hallazgos de la radiografía torácica, se sospechó de una infección por SARS-CoV-2. Con base en la ley rumana, la autopsia estaba indicada, porque no se realizó examen alguno de hisopado nasofaríngeo.

Se obtuvo consentimiento informado por escrito de la madre para realizar la autopsia y publicar los resultados científicos. La madre declaró que no visitó el hospital para el seguimiento pre- ni posnatal, y que no se adhirió al esquema de vacunación programado. El rastreo de contactos no identificó un posible contacto con la infección por SARS-CoV-2, pero fue difícil obtener un historial médico completo de la madre.

### Hallazgos en la autopsia

Para reducir el riesgo infeccioso, un técnico capacitado, junto con el jefe del Servicio de Patología (GS), realizó el examen post mortem en la sala de autopsias. Ambos usaron equipo de protección personal completo.

El examen externo mostró una bebé con piel extremadamente pálida, quien parecía haberse desarrollado normalmente (altura: 88 cm; peso: 10 kg), pero mostraba edema generalizado sin fóvea. Durante el examen in situ de los órganos se describió un timo normal, efusión pleural bilateral (50/50 ml) y pericardial (30 ml), hepatomegalia (536 g), esplenomegalia (64 g) y neumatosis intestinal.

**Fig. 2:**
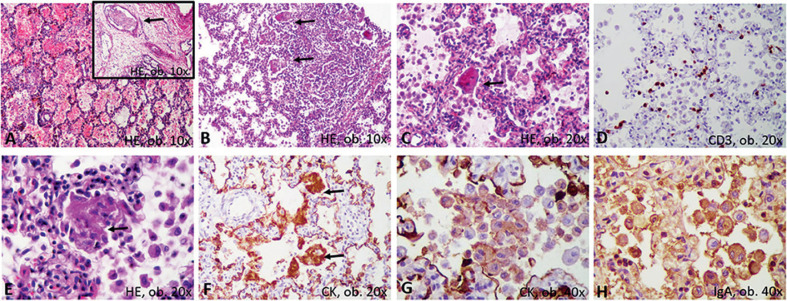
Características microscópicas e inmunohistoquímicas de la neumonía intersticial descamativa: (**A**) hemorragias y trombos (flecha); (**B**) daño bronquiolar; (**C**) neumocitos descamados, con cuerpos parecidos a un sincitio; (**D**) escasas células T; (**E**) necrosis de las células descamadas, con génesis de membranas; (**F**) recubrimiento de septos alveolares por membranas positivas a la citoqueratina; (**G**) positividad de células descamadas para la citoqueratina; (**H**) positividad para IgA en las células descamadas.

Ambos pulmones se hallaban agrandados, firmes y distelectásicos (136 g izquierdo; 169 g derecho), y sin áreas friables; el corte transversal no mostró rasgos macroscópicos sugerentes de bronconeumonía (Figura 1). Bajo el microscopio, el examen de las muestras pulmonares mostró daño alveolar difuso (DAD) bilateral, con descamación grave de los alveolocitos tipo II (neumocitos) y la presencia de macrófagos intraalveolares (Figura 2). Se demostró una positividad sincrónica al CD68/IgA/citoqueratina AE1/AE3 (CK) entre las células descamadas, sin expresión inmunohistoquímica (IHQ) de los marcadores de histiocitosis de Langerhans, CD1a y S100 policlonal. También se observó una escasa infiltración mononuclear, con predominio de linfocitos. El epitelio bronquial tenía daño focal y expresó positividad para IgA. Se encontró bronquiolitis descamativa asociada. También se describieron focos pequeños de eritrocitos extravasados, junto con trombos vasculares (Figura 2).

**Fig. 3:**
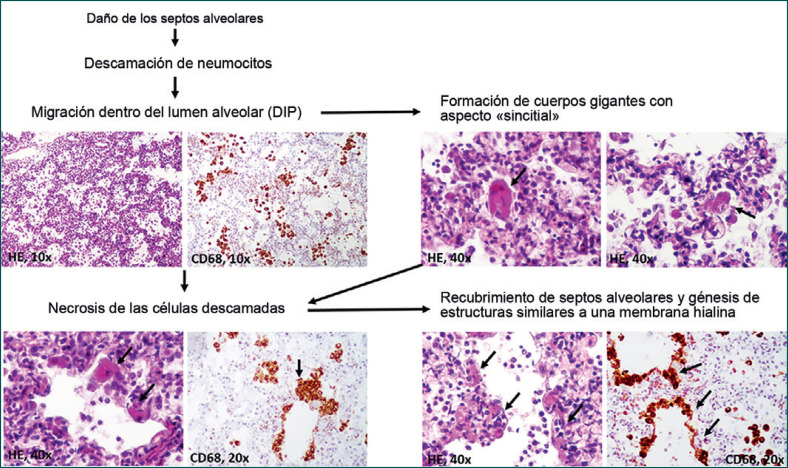
Génesis paulatina de estructuras similares a una membrana hialina en la neumonía intersticial descamativa (NID).

Un aspecto particular se refiere a la posibilidad de formación paulatina de membranas hialinas a partir de neumocitos descamados positivos para CD68/CK (Figura 3). Éstos se observaron predominantemente dentro de los alveolos, junto con macrófagos. La mayoría de los neumocitos descamados se observaron como células uninucleadas, redondas o elongadas. Algunos se encontraban agrandados, mostrando citoplasma eosinofílico, y los núcleos aparecían parcialmente desplazados hacia la periferia. Semejaban «células sincitiales». Por otro lado, dentro de los septos alveolares observamos algunos neumocitos agrandados, necróticos, positivos para CD68/CK, la fusión de los cuales llevó a la génesis de estructuras similares a una membrana hialina, positiva para CD68/CK, con recubrimiento de los septos alveolares. Dentro de los alveolos, había pocos neutrófilos en las membranas aledañas (Figuras 2, 3).

Dos muestras de tejido fresco de las áreas pulmonares distelectásicas se transportaron al Instituto Nacional Cantacuzino de Desarrollo e Investigación en Microbiología e Inmunología (Rumania), para realizar un ensayo de PCR en tiempo real. Aunque las características histológicas podrían corresponder al SARS, el ensayo de PCR no confirmó la participación del SARS-CoV-2 en las lesiones pulmonares antes descritas. Los exámenes moleculares realizados en el Instituto Nacional para Enfermedades Infecciosas Matei Bals (Bucarest, Rumania) en tejido pulmonar fijado en formalina e incluido en parafina tampoco lograron detectar por PCR al SARS-CoV-2, ni a los virus de influenza A o B, ni al virus sincitial respiratorio (VSR).

**Fig. 4:**
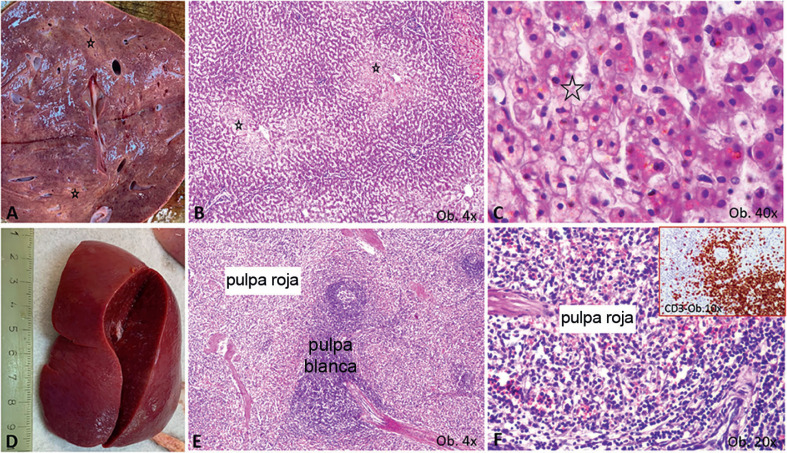
Características macro- y microscópicas del hígado y el bazo. (**A–C**) alteración grasa centrolobulillar del hígado, con área pálida macro- (**A**) y microscópica (**B**), la cual muestra un aspecto multivacuolar a mayor aumento (C), donde aparece marcada con *; (**D–F**) el espécimen de esplenomegalia está caracterizado por agrandamiento de la pulpa blanca (**E**) con un aspecto rico en células T en la pulpa roja (**F**), demostrado por la positividad a CD3 (esquina en **F**).

Con respecto a los otros órganos, el edema cerebral grave (960 g de peso) se asoció con hiperemia meníngea y la dilatación de los ventrículos laterales, sin signos de meningitis o encefalitis. En el hígado, agrandado, se observó degeneración vesicular bajo el microscopio. En el bazo, también agrandado, se describió hiperplasia leve de la pulpa blanca junto con infiltración de células T positivas a CD3 en la pulpa roja (Figura 4). Se observó hiperplasia de células T del área paracortical en las linfadenopatías mesentéricas pericecales. No se identificaron malformaciones cardiacas o de otro tipo, y tampoco miocarditis.

**Fig. 5:**
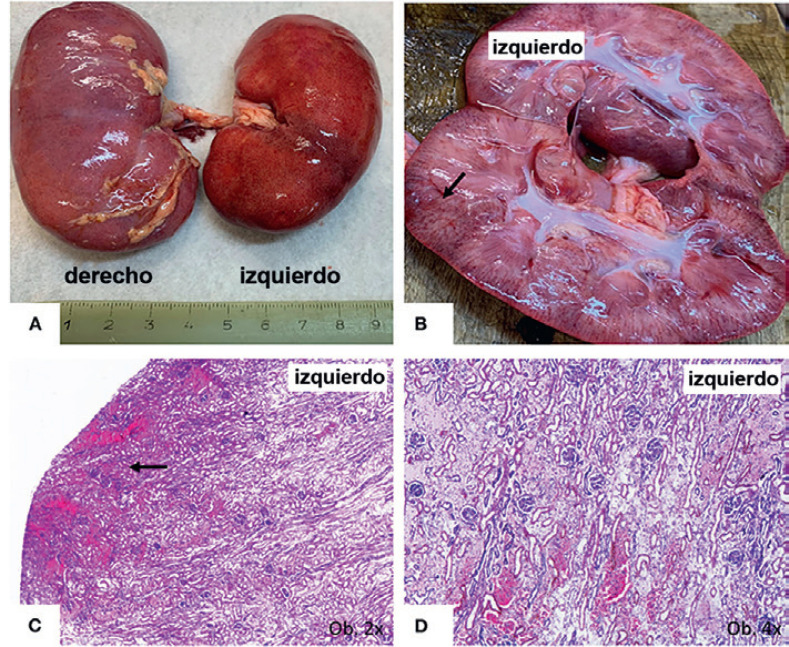
Hallazgos macroscópicos en los riñones. (**A**) Agrandamiento bilateral, con superficie hiperémica en el riñón izquierdo; (**B**, **C**) hiperemia pasiva de la corteza; (**C**, **D**) congestión parenquimatosa, con edema intersticial.

**Fig. 6:**
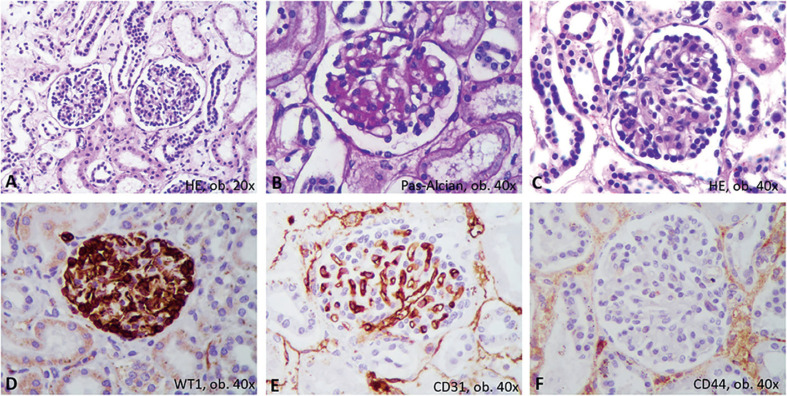
Glomerulonefritis proliferativa. En hematoxilina-eosina, las características microscópicas consisten en un agrandamiento del mesangio y proliferación de podocitos (**A–C**). Inmunohistoquímicamente, los podocitos proliferados fueron marcados con WT1 (**D**) y rodean a los capilares positivos para CD31 (**E**), sin positividad para CD44 (**F**).

El inusual agrandamiento bilateral de los riñones (ambos pesaron 146 g) requirió un examen más detallado. Macroscópicamente, el riñón izquierdo se encontraba claramente hiperémico, con predominio de hiperemia cortical en el corte transversal (Figura 5). Bajo el microscopio, la mayoría de los glomérulos mostraron agrandamiento del mesangio, con almacenamiento de células positivas para IgA y la proliferación de podocitos positivos para WT1, sin modificaciones en las células de la cápsula de Bowman; este hecho se confirmó por tinción negativa para CD44 (Figura 6). También se describió nefritis intersticial con células mononucleares. La corteza, hiperémica a simple vista, mostró dilatación de las venas bajo el microscopio (Figura 5), posiblemente por efecto de un flujo sanguíneo deficiente.

Con base en los aspectos morfológicos, histológicos e IHQ, el diagnóstico post mortem fue «NID asociada con glomerulonefritis proliferativa por IgA, posiblemente ocurrida en el contexto de una infección similar al SARS-CoV». La muerte fue resultado del síndrome de fallo multiorgánico (SFMO), que se inició probablemente por una lesión pulmonar grave.

## Discusión

Los primeros dos casos de NID asociada con insuficiencia renal crónica en niños se publicaron en 1992 [4]. En uno, la NID se diagnosticó a los 10 meses con base en una biopsia de pulmón, con ocurrencia de proteinuria a los 5 años de edad, y de glomeruloesclerosis a los 16, que requirió hemodiálisis [4]. El tercer caso de NID idiopática con manifestaciones extrapulmonares se publicó en 2014 y se presentó en una niña de 30 meses de edad [2]. No identificamos otros casos similares en infantes.

En tiempos anteriores al COVID, la NID con glomerulonefritis por IgA con rápido deterioro en un infante con anemia grave, deshidratación y mala alimentación, como en el caso presente, se habría considerado una presentación pediátrica infrecuente [2]. Sin embargo, en la pandemia de COVID-19, la rápida descompensación pulmonar − a pesar de la ausencia de antecedentes epidemiológicos, que fueron difíciles de obtener con base en el historial médico de la familia − nos forzó a incluir el caso entre los «sospechosos».

La pandemia de COVID-19 ha afectado a millones de personas en todo el mundo, pero los casos en niños son raros. Más aún, en los pocos casos reportados en la literatura, 90% de los niños positivos para COVID tuvieron una forma más leve de la enfermedad que los adultos, y las muertes fueron extremadamente raras [5, 6]. El espectro clínico recuerda al de la influenza, e involucra fiebre, tos, estornudos, dolor de garganta, fatiga y mialgia [7, 8, 9]. Presentaciones atípicas con manifestaciones gastrointestinales, cómo vómito y diarrea, así como desnutrición, se han descrito también en niños [9]. Se ha señalado una baja saturación de oxígeno (<92%) en menos de 5% de los casos, en contraste con la taquipnea y la taquicardia, que estuvieron presentes en proporciones sustanciales (28.7 y 42.1%, respectivamente) al momento del ingreso hospitalario [9]. Aun cuando el COVID-19 no se confirmó en el caso anterior, nuestra paciente presentó tos y fiebre, además de dificultad respiratoria grave y baja saturación de oxígeno, lo que sugirió la posibilidad de un mal desenlace.

Otro padecimiento poco frecuente que debe considerarse para el diagnóstico diferencial de tales casos es el conocido como síndrome multisistémico inflamatorio pediátrico temporalmente asociado con SARS-CoV-2 (PIMS-TS) [10, 11, 12]. Se han registrado casos raros en Europa y Norteamérica, con características similares a otros padecimientos inflamatorios pediátricos, como la enfermedad de Kawasaki, la sepsis bacteriana, el síndrome de choque tóxico o el síndrome de activación macrofágica. Se sabe que el PIMS-TS está asociado con disfunción miocárdica, lesiones en las arterias coronarias y afectación gastrointestinal y sistémica [10]. De acuerdo con el Colegio Real de Pediatría y Salud Infantil, debe sospecharse de PIMS-TS en cualquier niño que presente fiebre persistente, signos de inflamación, entre ellos neutrofilia, niveles aumentados de proteína C-reactiva y linfopenia, y de disfunción en uno o varios órganos, como choque y afecciones cardiacas, respiratorias, renales, gastrointestinales o neurológicas [11, 12]. Nuestra paciente presentó fiebre, pero la madre no pudo precisar el tiempo de inicio de la fiebre, y por tanto no fuimos capaces de delinear su persistencia. A pesar de la presencia de un padecimiento respiratorio y daño renal, como se demostró en la autopsia, tanto la cuenta de neutrófilos como la de linfocitos se encontraban en intervalos normales, y los cultivos de sangre y orina fueron negativos, como lo fue también la prueba de PCR para SARS-Cov-2, que puede ser positiva o negativa en el PIMS-TS. En términos de estudios de imagen y del ECG, el PIMS-TS se asocia con miocarditis, valvulitis, efusión pericárdica, dilatación de las arterias coronarias, infiltrados simétricos dispersos y efusión pleural en la radiografía, además de linfadenopatías, ascitis, hepatoesplenomegalia o signos de colitis e ileítis en el ultrasonido abdominal. En nuestro caso, ni el ECG ni el ultrasonido abdominal produjeron hallazgos patológicos, que se confirmaron por la autopsia. Adicionalmente, los hallazgos en la radiografía torácica no fueron sugerentes de PIMS-TS, puesto que los infiltrados dispersos fueron asimétrico y el pulmón derecho estaba completamente opacificado, mientras que se identificaron infiltrados dispersos en el pulmón izquierdo. Con base en estos hechos, consideramos poco probable que los síntomas de nuestra paciente se relacionaran con el PIMS-TS.

En términos de los parámetros de laboratorio, los niños con COVID-19 parecen mostrar tasas de linfopenia relativamente más bajos, y biomarcadores inflamatorios más altos, en comparación con los adultos [5]. De esta forma, una revisión que incluyó a 66 niños de 12 estudios reveló cuentas leucocitarias normales en 69.2% de los casos, neutropenia en 6%, neutrofilia en 4.6% y linfopenia en 3%. Sin embargo, la proteína C-reactiva se encontró elevada solamente en 13.6% de estos niños [13]. Un valor levemente elevado de la proteína C-reactiva se detectó también en nuestro caso.

En niños con COVID-19, los hallazgos de la radiografía torácica suelen ser inespecíficos [14]. Se observó la afectación multilobular y una distribución periférica de las lesiones pulmonares, junto con la consolidación y un halo circundante. Las opacidades con aspecto de vidrio molido, más obvias en la TC torácica, se observaron en un tercio de los niños [9, 14].

Con respecto a la imagen histológica del pulmón, se correspondió con los pocos casos reportados de COVID-19, en los que la positividad se demostró mediante un ensayo de PCR en tiempo real [6, 15]. De manera similar a nuestro caso, en autopsias de pacientes adultos con COVID-19 se describen daños graves al parénquima pulmonar, con infiltración de células mononucleares en los septos alveolares, descamación de neumocitos y la génesis de una membrana hialina en el contexto del síndrome de dificultad respiratoria aguda (SDRA) [6, 15].

Las células gigantes, multinucleadas y positivas para CD68 observadas en nuestras muestras se denominaron «células sincitiales multinucleadas» en los casos positivos de COVID-19 [15]. Los neumocitos elongados se consideraron «alteraciones parecidas a efectos citopáticos virales» [15].

Aunque se creía que la evolución clínicamente desfavorable de los pacientes con COVID-19 se relacionaba con el SDRA, se ha reportado una evolución incluso peor que en pacientes con SDRA «clásico». El presente caso podría ofrecer una nueva explicación para la falta de respuesta a la terapia con oxígeno en casos similares. Se sabe ya que, luego de una lesión, la denudación de la membrana basal alveolar puede inducir descamación de células epiteliales/neumocitos positivos para CK en los alveolos [16]. Sin embargo, aún se entiende poco la evolución paulatina después de la descamación.

Se cree que los neumocitos descamados están involucrados activamente en la restauración de los septos alveolares, o incluso inducen fibrosis como resultado de la transición epitelial-mesenquimal [16]. Aunque uno de nuestros estudios previos en pacientes con SDRA mostró que las membranas hialinas pueden expresar CD68 simultáneamente [17], la génesis de estas membranas no se ha elucidado. En aquellos casos, las membranas se hallaban bien estructuradas, y no examinamos las etapas iniciales de su génesis. Por esta razón, consideramos que estaban formadas por material hialino y que inducían la falta de respuesta a la terapia con oxígeno. En el presente caso, el paradigma consistió en la génesis inusual de las membranas. Con base en el examen histológico de secciones pulmonares con cortes múltiples y la positividad de las células y membranas para CD68/CK, demostramos que los neumocitos pueden necrosarse e inducir el recubrimiento de los septos alveolares (Figura 3). En el siguiente paso, es muy probable que se transformen en el material hialino. Si esto se demostrara en otros casos o en estudios experimentales, podría abrir nuevos caminos para el manejo terapéutico de la NID semejante al SARS-CoV.

La pulpa roja del bazo rica en células T y la hiperplasia de células T de los nodos linfáticos mesentéricos demostró, como en el COVID-19, la hipótesis de una respuesta inmune mediada por linfocitos T en este caso [15].

Con respecto a los otros órganos, en adultos positivos para COVID-19, el examen post mortem reveló una leve alteración grasa en el hígado y un infiltrado inflamatorio dentro de los espacios portales [15]. En nuestro caso, la hepatomegalia se asoció con una alteración grasa centrolobulillar inducida por hipoxia, que se corresponde con los cambios morfológicos previamente reportados. La infección por SARS-CoV-2 también puede inducir cambios no-significativos en el miocardio, como infiltrados leves de células mononucleares [15], pero esto no es un hecho [6, 15]. En niños, se ha descrito que la evolución fatal es inducida por una coagulación anormal, encefalopatía e insuficiencia cardiaca y renal aguda, que se confirmaron en la autopsia [7, 8]. En nuestro caso, la insuficiencia renal fue inducida por la glomerulonefritis por IgA, y la hiperemia pasiva podría ser resultado de un deterioro en el flujo sanguíneo por un posible trombo.

La baja incidencia de COVID-19 en niños se explica por la inmadurez del sistema inmune, junto con la presencia simultánea de otros virus en la mucosa de las vías respiratorias superiores e inferiores, que podrían obstaculizar el crecimiento del SARS-CoV-2 [18, 19, 20]. En el caso presentado en este artículo, la influenza y la infección por VSR también quedaron excluidas. Debido a las diferencias en la expresión de la enzima convertidora de angiotensina o a la falta de síntomas, la glomerulonefritis en este grupo etario puede quedar sin diagnóstico y confundirse clínicamente con signos de desnutrición [18, 19, 20].

Este caso destaca las dificultades diagnósticas que implica la evaluación de niños con padecimientos respiratorios, particularmente en la era de COVID-19. También pone de manifiesto la importancia de la autopsia en tales casos.

## Limitaciones del estudio

Una limitación de este caso es que no realizamos pruebas serológicas para COVID-19, puesto que se reconoce que aun si el PCR es negativo, la evidencia de seroconversión puede sugerir la infección.

## Conflictos de interés

Los autores declaran que la investigación se condujo en ausencia de cualquier relación comercial o financiera que pudiera considerarse un potencial conflicto de interés.

## Información de licencias

^©^ 2020 Simona Gurzu, Catalin Bogdan Satala, Lorena Elena Melit, Adrian Streinu-Cercel, Dan Otelea, Brandusa Capalna, Claudiu Ioan Puiac, Janos Szederjesi, Ioan Jung: COVID-19 Like Findings in a Fatal Case of Idiopathic Desquamative Interstitial Pneumonia Associated With IgA Glomerulonephritis in a 13-Month-Old Child. Front Pediatr. 2020; 8: 586666. (traducción; declaración de disponibilidad de datos, declaración ética, contribución de los autores y agradecimientos acortada), con licencia bajo CC BY 4.0 (https://creativecommons.org/licenses/by/4.0/deed.es).

## Figures and Tables

**Tabla 1:** Los parámetros sanguíneos son indicativos de anemia grave, desnutrición y deshidratación

Parámetro	Valor de la paciente	Intervalo normal
Leucocitos (×10^3^/µL)	8.21	4–12
Hemoglobina (g/dL)	***1.5***	10–14.2
Eritrocitos (×10^6^/µL)	***1.27***	3.5–4.5
Hematocrito (%)	***6.7***	36–47
Volumen celular medio − VCM (fL)	***52.8***	80–98
Neutrófilos (×10^3^/µL)	3.06	1.5–8.5
Linfocitos (×10^3^/µL)	4.42	3–10.5
Proteína C-reactiva − CRP (mg/L)	***14.6***	<5
Urea (mg/dL)	***39.80***	10.51–35.55
Creatinina (mg/dL)	***0.36***	0.57–1.11
Aspartato aminotransferasa − AST (U/L)	36	5–34
Alanina aminotransferasa − ALT (U/L)	25	0–55

Las cifras en negritas cursivas representan valores anormales (modificados), ya sea por encima o por debajo del intervalo normal.
